# Artificial Intelligence Must Be Made More Scientific

**DOI:** 10.1021/acs.jcim.4c01091

**Published:** 2024-07-27

**Authors:** Peter V Coveney, Roger Highfield

**Affiliations:** † Centre for Computational Science, Department of Chemistry, University College London, London WC1H 0AJ, U.K.; ‡ Advanced Research Computing Centre, University College London, London WC1H 0AJ, U.K.; § Institute for Informatics, Faculty of Science, University of Amsterdam, 1098XH Amsterdam, The Netherlands; ∥ Center for Advanced Studies, Ludwig Maximilian University of Munich, D-80539 München, Germany; ⊥ Science Museum, Exhibition Road, London SW7 2DD, U.K.; ○ Sir William Dunn School of Pathology, University of Oxford, Oxford OX1 3RE, U.K.; ▽ Department of Chemistry, University College London, London WC1h 0AJ, U.K.

## Abstract

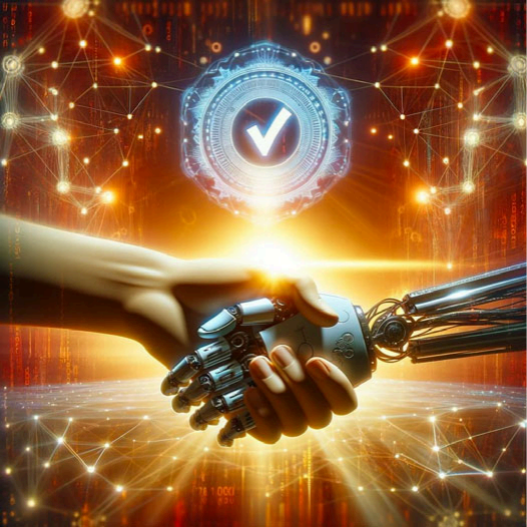

The role of AI within science is growing. Here we assess its impact on research and argue that AI often lacks reproducibility, transparency, objectivity, and mechanistic understanding. To ensure AI benefits research, we need to develop forms of AI that are fully compatible with the scientific method.

Artificial Intelligence is taking hold in science, even though it remains a long way from living up to the wilder claims of media headlines. But is it changing what we mean by science? The answer is an emphatic “no”: the current generation of AI is in many respects not even scientific.

The definition of precisely what we mean by science attracts debate among philosophers and historians, but there is general agreement that it is a fusion of observation and reason: radical empiricism (data without reason) and rationalism (reason without data) were rejected centuries ago, and instead scientists place their faith in using theory to make predictions and prompt new experiments, conducting experiments to produce data to shape theory, and so on.^1^ A premium is placed on reproducibility, ensuring science is objective, which sets it apart from other human endeavors.

Centuries ago, Bacon described how scientists nurture this symbiosis between rationalism and empiricism, likening them to bees.^2^ With the rise of the computer, another kind of science has taken hold, where simulations make actionable predictions. Mathematical models that capture our understanding of the atmosphere and oceans, when combined in a computer with data from satellites, weather stations, and so on, enable forecasting to save lives. The most dramatic forward-looking examples are digital twins of the human body.^3^

Now we are entering a new era of computation, with AI growing in importance. Yet few remember earlier hype cycles, where AI bust followed boom. We have also lost sight of how the abilities of our 20-W brains remain spectacular, even compared with exascale computers that draw a million times more power. It is an embarrassing fact that there is no widely accepted definition of natural intelligence,^4^ so what exactly do we mean by AI? We have put excessive faith in computers.^5^

Despite these shortcomings, bold, not to say outlandish, claims about AI are being made by giant US-based tech companies.^6^ They have one overriding motivation: to make money. Big institutions are scrambling to adopt AI for fear of missing out. Governments are only too happy to jump on the bandwagon, with the promise that AI will make them more persuasive to their electorates, as well as more effective and efficient.

By claiming that computer algorithms can transcend human intelligence, their most feverish acolytes believe that machines can take over many functions of humans. Ironically, some of the wilder claims come from companies that rely on legions of crowd-sourced labor, what Jeff Bezos called ‘artificial artificial intelligence’,^7^ or “pseudo-AI”, to help AI do menial but tricky tasks.

There is a lazy assumption that AI can “do science” too, but machine learning methods were and still are pattern spotters designed to solve technological problems. Their origins have far more to do with the intelligence and security communities devising ways to get computers to sift through oceans of digital data than scientists trying to understand nature.

Here AI certainly does have a role. Perhaps the best-known example is the protein structure prediction model AlphaFold,^8^ which has mapped the universe of every known protein. For molecular biologists, AlphaFold is a very fast alternative to X-ray crystallography. Like so much machine learning, AlphaFold works best on what it has been trained to see. But, being a glorified look-up table, it remains hard to say when it will work reliably and when it may fail. Put another way, it is very hard to quantify its uncertainties.

Another fashionable topic is the use of AI to generate machine-learned interaction potentials (MLIPs), for example, ones which can be used to perform classical molecular dynamics simulations. Determining the form of these force fields, or potential parametrizations, is tedious, so, when new ones are required, it is proposed that AI learn how to map from atomic properties to molecular potential energies and/or other quantities of interest, from as large a data set as possible.^9^ This results in a neural network with many hundreds of thousands of fitting parameters, which are the connection weights between pairs of “neurons” in the network. Again, attempting to quantify the uncertainties in such MLIPs is difficult for two reasons: there are far too many parameters, and they are merely fitting parameters with no intrinsic physicochemical meaning.

In fact, we do understand the science of molecular interactions, so one can also use a physics-based interaction potential (with terms which are scientifically meaningful) with perhaps hundreds to a few thousand parameters. We have been able to use new, scalable forms of uncertainty quantification which allow us to show that typically only between ten and 20 of these force field parameters have a significant influence on the properties of interest.^10^ In other words, we acquire real insights and understanding about what parameters are important.

By comparison, we cannot make sense of what is going on inside an MLIP or AlphaFold, which require hundreds of thousands to hundreds of millions of parameters. They represent at once the strength and weakness of AI. While the astronomical number of parameters explains why ML can successfully fit so many arbitrary relationships, it also accounts for their unreliability and their failure to provide satisfactory scientific explanations.

Moreover, they are typically trained on some selected data set, broken into a larger portion for training and a smaller subset for validation. But would they work on a different data set? Often, they fail because they are then extrapolating rather than interpolating.

Generative methods suffer from similar problems but are more strongly dependent on random number generators, so *a fortiori*, they produce different answers each time the code is run. This is reminiscent of molecular dynamics where one-off simulations are not reproducible.^11,12^ There are other challenges to reproducibility,^13^ which require access to the underlying data and ML algorithms employed, which may be kept confidential,^14,15^ and sometimes access to substantial computational power.

While science seeks understanding, AI rests on statistical inference. This does not make it wrong *per se*, but recall the old saw that correlation is not causation. Using results from ergodic, Ramsey, and algorithmic information theories, one can show that large databases contain arbitrary numbers of correlations that increase rapidly with the amount, not the nature, of the data. These correlations also arise in large, randomly generated databases, implying that most are spurious.^16^ Sifting false correlations from the true requires the scientific method.

Although computers create a veneer of objectivity, humans still play a central role in how AIs are set up and used. Most of the time, to train an AI you must define the categories into which the AI will sort its answers. But any such classification is arbitrary and riddled with ambiguities, reflecting the developers’ own motivations: human bias is baked into AI, even *before* training.

AI typically rests on various assumptions that also reflect human choices, rather than are based on science. Essentially all ML algorithms assume smooth (differentiable) relationships between the quantities involved in its internal data analysis. These are made purely for convenience in order to permit the use of linear algebra, standard software libraries, and substantial speed-up by GPU accelerators. Nonetheless, AI and ML most certainly do provide a wide range of nonlinear predictions. They do this because, though linear algebra is to the fore, they include nonlinear activation functions which relate input to output data.

The comforting assumption that we live in a differentiable world might suggest that sacrificing a little accuracy by moving from double to half precision and even quarter precision in the context of floating-point numbers makes little difference, or that the bell curve of Gaussian statistics is omnipotent. In the real world, none of this holds in general. Sharp discontinuities occur, a hallmark of nonlinear behavior.^17^

Ultimately, the world is highly nonlinear, and because nonlinear science is counterintuitive and often nondifferentiable, there is a temptation to ignore it. Perhaps the ultimate manifestation of nonlinearity, which is rarely discussed, is how rounding may generate profound errors in digital computers.^18,19^

Though misguided, one can understand why some scientists welcome AI as an alternative to Bacon’s busy bees: in complex fields such as the biological sciences, AI’s focus on answers rather than understanding is seductive. But, when extended to healthcare, for example, it is vital we understand how proposed therapies work and free them of intrinsic biases—not just in terms of the unrepresentative nature of the data they are trained on, but how AIs are built in the first place.

There is growing excitement in some quarters about the new wave of AI “foundation models,” based on “general-purpose AI” or “GPAI” systems that can supposedly solve scientist’s problems via an interactive LLM interface akin to ChatGPT. Examples of these “AI4Science” models include DiG (Digital Graphormer)^20^ for molecular distributions, MatterGen^21^ for inorganic materials design, and TamGen (target-aware molecule generation).^22^

As these models rain down on us, rather than surrendering the very bastion of science, it is time scientists demanded that AI/ML conform with the highest standards of scientific inquiry. We need a focus on reproducibility and, above all else, on theoretical concepts and methods that provide mechanistic insight and understanding.

AI undoubtedly offers considerable benefits to science, but we must never turn our backs on the reproducible blend of rationalism and empiricism that has endured for three centuries. One way forward might be Explainable AI (xAI), and we should embrace “causal AI”, as long as AI can explain its inner workings and predictions in scientific terms. Another is “Big AI,” a combination of machine learning and physics-based methods, which constrain AI to obey the laws of nature.^23^ In such contexts, their strengths and weaknesses are complementary, and it may make sense to couple them, for instance, in drug discovery.^24^

Science is among the most precious of humanity’s creations, and it needs defending and articulating more than ever. Bacon’s bees are under threat from AI, when they need to thrive. AI must conform to the scientific method.

## References

[ref1] Coveney P. V.; Dougherty E.; Highfield R. R. Big data need big theory too. Philos. Trans. R. Soc. A 2016, 374, 20160153 10.1098/rsta.2016.0153. PMC5052735 27698035

[ref2] Bacon F.; Hutchins R. M.; Adler M. J. Novum organum. In Great Books of the Western World*;* Hutchins R. M., Adler M. J., Eds.; Encyclopædia Britannica, 1952; Vol. 35.

[ref3] Coveney P. V.; Highfield R. R. Virtual You: How Building Your Digital Twin Will Revolutionize Medicine and Change Your Life*;* Princeton University Press, 2023.

[ref4] Hampshire A.; Highfield R. R.; Parkin B. L.; Owen A. M. Fractionating Human Intelligence. Neuron 2012, 76, 1225–1237. 10.1016/j.neuron.2012.06.022. 23259956

[ref5] Coveney P. V.; Highfield R. R. When we can trust computers (and when we can’t). Philos. Trans. R. Soc. A 2021, 379, 20200067 10.1098/rsta.2020.0067. PMC8059589 33775149

[ref6] Crawford K. Atlas of AI; Yale University Press: New Haven and London, 2021.

[ref7] Pontin J. Artificial Intelligence, With Help From the Humans, New York Times, March 25, 2007.

[ref8] Jumper J.; Evans R.; Pritzel A.; Green T.; Figurnov M.; Ronneberger O.; Tunyasuvunakool K.; Bates R.; Žídek A.; Potapenko A.; Bridgland A.; Meyer C.; Kohl S. A. A.; Ballard A. J.; Cowie A.; Romera-Paredes B.; Nikolov S.; Jain R.; Adler J.; Back T.; Petersen S.; Reiman D.; Clancy E.; Zielinski M.; Steinegger M.; Pacholska M.; Berghammer T.; Bodenstein S.; Silver D.; Vinyals O.; Senior A. W.; Kavukcuoglu K.; Kohli P.; Hassabis D. Highly Accurate Protein Structure Prediction with AlphaFold. Nature 2021, 596, 583–589. 10.1038/s41586-021-03819-2. 34265844 PMC8371605

[ref9] Zhang S.; Makos M.łg. Z.; Jadrich R. B.; Kraka E.; Barros K.; Nebgen B. T.; Tretiak S.; Isayev O.; Lubbers N.; Messerly R. A.; Smith J. S. Exploring the frontiers of condensed-phase chemistry with a general reactive machine learning potential. Nat. Chem. 2024, 16, 727–734. 10.1038/s41557-023-01427-3. 38454071 PMC11087274

[ref10] Edeling W.; Vassaux M.; Yang Y.; Wan S.; Guillas S.; Coveney P. V. Global ranking of the sensitivity of interaction potential contributions within classical molecular dynamics force fields. npj Comput. Mater. 2024, 10, 87 10.1038/s41524-024-01272-z.

[ref11] Coveney P. V.; Wan S. On the calculation of equilibrium thermodynamic properties from molecular dynamics. Phys. Chem. Chem. Phys. 2016, 18, 30236–30240. 10.1039/C6CP02349E. 27165501

[ref12] Coveney, P.V.; Wan, S. *Molecular Dynamics: Probability and Uncertainty*; Oxford University Press, 2025.

[ref13] Pouchard L.; Reyes K. G.; Alexander F. J.; Yoon B.-J. A rigorous uncertainty-aware quantification framework is essential for reproducible and replicable machine learning workflows. Digital Discovery 2023, 2, 1251–1258. 10.1039/D3DD00094J.

[ref14] AlphaFold3 - why did Nature publish it without its code? Nature. 2024, 629, 728. 10.1038/d41586-024-01463-0 38778239

[ref15] Gibney E. Not all ’open source’ AI models are actually open: here’s a ranking. Nature 2024, 10.1038/d41586-024-02012-5. 38898259

[ref16] Calude C. S.; Longo G. The deluge of spurious correlations in big data. Found. Sci. 2017, 22, 595–612. 10.1007/s10699-016-9489-4.

[ref17] Succi S.; Coveney P. V. Big Data: the End of the Scientific Method?. Philos. Trans. R. Soc. A 2019, 377, 20180145 10.1098/rsta.2018.0145. PMC6388004 30967041

[ref18] Boghosian B. M.; Coveney P. V.; Wang H. A New Pathology in the Simulation of Chaotic Dynamical Systems on Digital Computers. Adv. Theory Simul. 2019, 2, 1900125 10.1002/adts.201900125. 34527854 PMC8427473

[ref19] Klöwer M.; Coveney P. V.; Paxton E. A.; Palmer T. N. Periodic orbits in chaotic systems simulated at low precision. Sci. Rep. 2023, 13, 11410 10.1038/s41598-023-37004-4. 37452044 PMC10349059

[ref20] Zheng S.; He J.; Liu C.; Shi Y.; Lu Z.; Feng W.; Ju F.; Wang J.; Zhu J.; Min Y.; Zhang H.; Tang S.; Hao H.; Jin P.; Chen C.; Noé F.; Liu H.; Liu T. Predicting equilibrium distributions for molecular systems with deep learning. Nat. Mach. Intell. 2024, 6, 558–567. 10.1038/s42256-024-00837-3.

[ref21] Zeni C.; Pinsler R.; Zügner D.; Fowler A.; Horton M.; Fu X.; Shysheya S.; Crabbé J.; Sun L.; Smith J. Mattergen: a generative model for inorganic materials design. arXiv 2023, 10.48550/arXiv.2312.03687. PMC11922738 39821164

[ref22] Xia Y.; Wu K.; Deng P.; Liu R.; Zhang Y.; Guo H.; Cui Y.; Pei Q.; Wu L.; Xie S. Target-aware Molecule Generation for Drug Design Using a Chemical Language Model. bioRxiv 2024, 10.1101/2024.01.08.574635. PMC11522292 39472567

[ref23] Coveney P. V.; Highfield R. Big AI: Blending Big Data with Big Theory to Build Virtual Humans. In Artificial Intelligence for Science*;* Choudhary A., Fox G., Hey T., Eds.; World Scientific, 2023; pp 381–398.

[ref24] Bhati A. P.; Wan S.; Alfè D.; Clyde A. R.; Bode M.; Tan L.; Titov M.; Merzky A.; Turilli M.; Jha S.; Highfield R. R.; Rocchia W.; Scafuri N.; Succi S.; Kranzlmüller D.; Mathias G.; Wifling D.; Donon Y.; Di Meglio A.; Vallecorsa S.; Ma H.; Trifan A.; Ramanathan A.; Brettin T.; Partin A.; Xia F.; Duan X.; Stevens R.; Coveney P. V. Pandemic drugs at pandemic speed: infrastructure for accelerating COVID-19 drug discovery with hybrid machine learning- and physics-based simulations on high-performance computers. Interface Focus 2021, 11, 20210018 10.1098/rsfs.2021.0018. 34956592 PMC8504892

